# Dogs do not use their own experience with novel barriers to infer others’ visual access

**DOI:** 10.1098/rspb.2023.2934

**Published:** 2024-06-12

**Authors:** Lucrezia Lonardo, Martina Putnik, Veronika Szewczak, Ludwig Huber, Christoph J. Völter

**Affiliations:** 1 Comparative Cognition, Messerli Research Institute, University of Veterinary Medicine of Vienna, Medical University of Vienna and University of Vienna, Vienna 1210, Austria; 2 Department of Comparative Cultural Psychology, Max Planck Institute for Evolutionary Anthropology, Leipzig 04103, Germany

**Keywords:** social cognition, dog cognition, goggles test, experience-projection test, theory of mind

## Abstract

Despite extensive research into the Theory of Mind abilities in non-human animals, it remains controversial whether they can attribute mental states to other individuals or whether they merely predict future behaviour based on previous behavioural cues. In the present study, we tested pet dogs (in total, *N* = 92) on adaptations of the ‘goggles test’ previously used with human infants and great apes. In both a cooperative and a competitive task, dogs were given direct experience with the properties of novel screens (one opaque, the other transparent) inserted into identical, but differently coloured, tunnels. Dogs learned and remembered the properties of the screens even when, later on, these were no longer directly visible to them. Nevertheless, they were not more likely to follow the experimenter’s gaze to a target object when the experimenter could see it through the transparent screen. Further, they did not prefer to steal a forbidden treat first in a location obstructed from the experimenter’s view by the opaque screen. Therefore, dogs did not show perspective-taking abilities in this study in which the only available cue to infer others’ visual access consisted of the subjects’ own previous experience with novel visual barriers. We conclude that the behaviour of our dogs, unlike that of infants and apes in previous studies, does not show evidence of experience-projection abilities.

## 1 Introduction

The capacity to think about others’ minds, and to understand that mental states cause behaviour, has been termed the ‘Theory of Mind’ (ToM [1]). It is advantageous to many social interactions and it is thought to be, at least in its richest definition, uniquely human [2]. Studying non-human animals, however, can help elucidate the nature and origin of some basic components of this ability [3]. Several studies have attempted to characterize non-human animals’, as well as pre-verbal human infants’, grasp of others’ mental states, in particular of others’ visual perspectives ([2,4–6] for reviews).

One of the main difficulties of non-verbal tests of ToM has been the problem of distinguishing between the subjects’ ability to infer higher-order (unobservable) mental states and their ability to reason about first-order representations of (perceptually available) behavioural cues [7,8]. That is, these tests usually cannot conclusively disambiguate between subjects using a set of behavioural rules that allow them to react appropriately to a test situation and subjects that attribute intentions and knowledge states to others. In order to evaluate whether an agent can see a target item, for instance, ‘behaviour readers’ might resort to a set of behavioural rules such as ‘the agent will approach the item if her eyes are open and her body is oriented toward the target’. Behaviour reading is purely based on elements that an external observer can directly see, without having to make inferences about mental representations. So-called ‘mind readers’, in contrast, would represent an intervening variable [9], in this case, the mental state of ‘seeing’. The mind reader will take the perspective of the agent and project her own experiences concerning the environment onto the agent. This will allow the mind reader to infer what the agent can currently see. If she infers that the agent can see the target, she might, in turn, attribute knowledge to the agent about the target’s location which the agent might take into account in the future when acting. However, as mentioned before, the majority of the tasks employed to test ToM non-verbally fail to address this important distinction between mind and behaviour reading (for a more detailed description of the issue, see e.g. [10–13]). To overcome this conceptual limitation, Heyes [14] proposed the ‘goggles test’ as a means to test an animal’s mind reading through experience projection—using the own experience with visual barriers, the goggles, to infer what others could see. These goggles appear identical from the outside, except for their colour, but one is see-through and the other opaque. The animal would first experience these differing properties of the two goggles. In the test, they would see another agent wearing one of the two goggles. If the animals behaved as if they understood that one of the goggles would obstruct someone else’s line of sight but the other would not, one could infer that the animals took into account others’ mental state of ‘seeing’ because behavioural cues are unavailable in this case.

The hypothesis that one of the mechanisms through which we can learn about others’ minds is by using our own experience as a model was tested with 18-month-old human infants. Meltzoff & Brooks [15] familiarized a group of infants to ‘trick blindfolds’ (instead of goggles). These appeared opaque but in reality, when worn close to one’s eyes, they were see-through. Infants who had experienced wearing the trick blindfolds themselves followed a blindfolded adult’s gaze to a toy more than infants who had experienced regular, fully opaque, blindfolds. These results were interpreted as evidence that we, humans, from an early age use our own experiences to learn about others’ mental states.

The goggles test was later adapted for chimpanzees (*Pan troglodytes* [16,17]). Karg *et al*. [16] implemented the test both within a gaze following and a stealing task. They made the apes experience visual barriers (‘face masks’ in the former task and lids of boxes in the latter task) that were either opaque or see-through from a certain perspective but that, at the moment of test, appeared equally opaque to the subjects. While the chimpanzees’ gaze-following behaviour was not affected by the property of the masks, their stealing attempts were preferentially directed at a reward under the lid that was opaque from an experimenter’s perspective. More recently, Kano *et al*. [18] showed in an eye-tracking study that great apes use their own experience with novel visual barriers to infer whether others can or cannot see through the same barriers. Specifically, in order to test their false belief understanding in a change-of-location task, apes were shown a video in which a human actor would observe from behind a barrier the hiding and displacement of an interesting object performed by a second actor. Prior to watching the same video, two groups of apes were given real-life experiences with the barrier behind which the actor would go hide in the video. One group of apes experienced the barrier as see-through from a close distance but opaque from far apart, while the other group of apes experienced the barrier as opaque both from up close and from a distance. The latter group visually anticipated that the human in the video would act according to a false belief about the location of the interesting object more than the group of apes who had experienced the barrier as being see-through from a close distance. These results are consistent with the apes attributing to the actor the mental states of ‘having seen’ or ‘not having seen’ the hiding and displacement of the object.

Despite some objections [10], experience-projection tests remain the best experimental tool currently available for distinguishing behaviour reading from mind reading [3,18,19]. Indeed, during the experience phase, at the very least, subjects have to learn about a psychological affordance: the barriers are opaque or transparent (i.e. they afford geometrical occlusion) only in relation to a visual system perceiving them from a certain perspective [15].

Outside of the primate lineage, dogs (*Canis familiaris*) represent an interesting species for the study of ToM abilities in non-human animals [20]. Indeed, they share an evolutionary history of close co-habitation with humans [21], which might make them appropriate models for the study of convergent evolution of socio-cognitive abilities [22–24]. Moreover, they show behaviour-reading skills when interacting with humans [20]. Specifically, converging evidence from different paradigms suggests that dogs have a good grasp of humans’ behavioural cues connected to ‘paying attention’ and ‘seeing’. Dogs behave as if humans were more likely to ‘register’ (i.e. perceive and remember [4,25]); events: when the humans’ eyes are open compared with closed [26]; when the humans orient towards rather than away from a target [26–34]; when the humans’ line of gaze is unobstructed compared with obstructed by different types of barriers [29,31,35–38] and when in light compared with when in darkness [39]. While dogs are known for their sense of smell, the aforementioned evidence of dogs’ perspective-taking abilities ([20], for a review) is almost exclusively based on tasks relying on visual stimuli, with a few exceptions using auditory cues. Many studies have even excluded the possibility that odour cues could affect task performance through appropriate controls.

To test whether dogs’ level I perspective-taking ability (the ability to represent others’ visual access [40,41]) rests on behaviour or mind reading, we confronted dogs in this study with two adaptations of the goggles test [14]. Previous studies, in which dogs discriminated between knowledgeable and ignorant experimenters, involved at least some, albeit sometimes subtle, behavioural differences between conditions. For example, in the guesser−knower task [42], the two informants between which dogs had to choose differed by covering their eyes or cheeks with their hands [31] or by their position in relation to the hider [28]. Also, in a false-belief task [43], the difference in knowledge states of the two informants concerning the location of hidden food differed owing to the timing when they were in the room and could thus witness the transfer of the food from the first to the second location [44].

In contrast, in the current instantiation of the goggles paradigm, dogs could not rely on different behavioural or contextual cues. They could only infer the experimenter’s visual access based on their own previous experience of seeing or not through two screens with differing properties, one being transparent and one being opaque. Crucially, in the test phase, dogs could not see the screens directly, as these were inserted in equally opaque tunnels. This differs from previous procedures where dogs could directly see the visual barriers (e.g. [35,37,38,45]).

## 2 Experiment 1

In experiment 1, we used a gaze-following task embedded in a foraging context. We expected the dogs to interpret an experimenter’s gaze cues as cooperative, communicative signals, as in previous studies on gaze following with dogs [46,47]. Following the results of Meltzoff & Brooks [15] with human infants and by Kano *et al*. [18] with great apes, we hypothesized that dogs too were able to project onto the experimenter their own previous experience of seeing or not seeing through novel screens. Hence, we predicted that they should have been more inclined to follow the experimenter’s gaze to a bucket potentially containing food when the experimenter’s view was unobstructed compared with when it was obstructed by an opaque screen.

### 2.1 Methods

Experiment 1 was pre-registered (https://osf.io/xsgbz/?view_only=7b5ef9487213421a921965732e5a4ffb). The sample size was determined based on previous studies using a similar procedure with human infants and dogs [15,47].

The procedures described in this study were discussed and approved by the Ethics and Animal Welfare Committee of the University of Veterinary Medicine of Vienna in accordance with the university’s guidelines for good scientific practice (ETK-151/10/2021 and ETK-162/09/2022).

#### 2.1.1 Subjects

The subjects were 32 pet dogs (23 females) of various breeds and mixes. Electronic supplementary material, table S1 reports their demographic information.

Eleven additional dogs were recruited but not included in the analyses owing to not passing a manipulation check pre-test (five dogs; see §1.1.3); being too tall for the set up (two dogs); video malfunction (three dogs) or their caregiver not following the instructions (one dog).

#### 2.1.2 Set up

**Figure 1:**
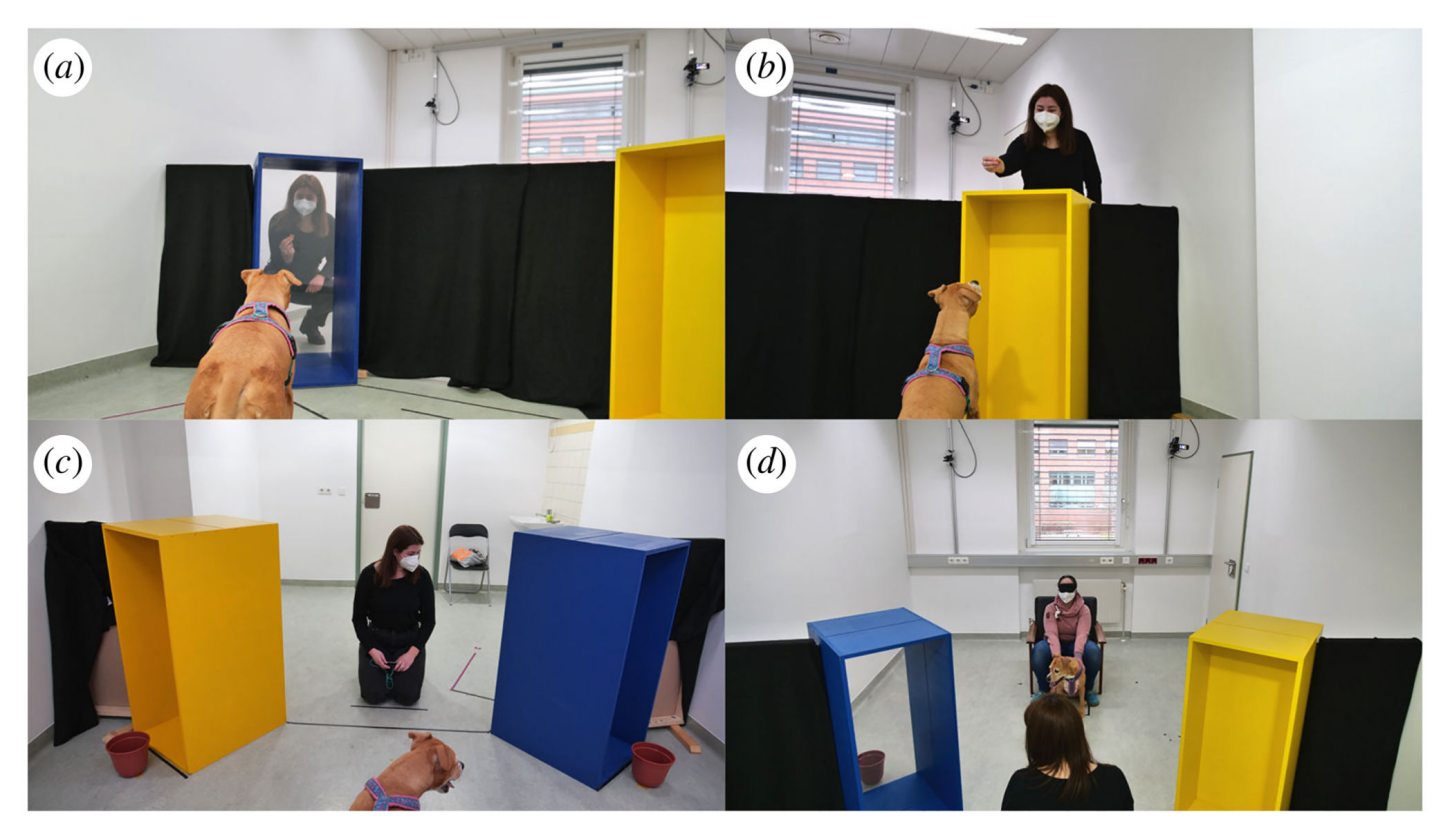
Experience (*a,b*) and test (*c,d*) phase of experiment 1. During the experience phase, the experimenter showed a piece of food both in front of (as in *a*) and above (as in *b*) each of the two tunnels, while the dog was on the ‘experimenter’s’ side of the room, i.e. the side where the experimenter was going to stay during test trials. During the test phase, from their starting position, dogs could not see the screens inside the tunnels (*c*) and were supposed to realize that the experimenter could only see one of the targets from her side of the room (*d*).

Experiments took place in a 6.05 × 3.33 m room, which was divided into two sides by a set of wooden barriers and two wooden tunnels (110 × 50 × 60 cm; figure 1). The tunnels appeared identical from the outside, except for their colour: one blue, the other yellow. One of the two tunnels contained a transparent screen. The other tunnel contained an opaque screen.

#### 2.1.3 Procedure and design

The whole procedure required a single session of approximately 30 min. During an introduction phase similar to the one used by Met *et al*. [47], upon entering the experimental room, dogs were left free to retrieve food contained in four brown plastic buckets, spread randomly across the room, while their caregivers received instructions. If dogs were too intimidated to do so spontaneously, they received encouragement from their caregiver and the experimenter. This procedure was repeated twice to ensure dogs approached the buckets and ate from them without hesitation, to establish a foraging context for the dogs.

##### 2.1.3.1 Experience phase

Next, during the experience phase, dogs had the opportunity to associate the colour or the location of each tunnel with the property of its screen (opaque or transparent). More details in the electronic supplementary material.

##### 2.1.3.2 Manipulation check phase

Before testing the dogs, we verified that the experience phase was effective, i.e. that, by the end of it, dogs remembered where the transparent screen was. To this end, dogs were held in their central starting position by their caregiver on one side (the dog’s side) of the room. From there (being 142 cm away from each tunnel), they could only see the tunnels but not the screen therein contained. On the other side of the room, the experimenter showed a chopping board and a piece of food, before sitting on the floor and hence disappearing behind the tunnels and barriers, in a position equidistant from both tunnels. The experimenter then started to produce chopping sounds with a knife on the chopping board and the dogs were released by their blindfolded caregivers. We reasoned that dogs would have been motivated to watch the experimenter and hence, if they remembered which tunnel contained the transparent screen, they should have approached it first, as this was the only area in the room that would have granted them visual access to the experimenter. The manipulation check trial lasted 90 s, at the end of which the dogs received a piece of food irrespective of what they had done. If the dogs did not look through the transparent screen during the entire manipulation check trial, the experimenter showed again a piece of high-value food behind both screens and repeated the manipulation check a maximum of once. If, again, dogs did not look through the transparent screen, they were excluded from the study.

##### 2.1.3.3 Test phase

After the manipulation check, during test trials, the experimenter knelt in front of the dogs on one side of the room (the experimenter’s side) without the central visual barrier, so that she was now fully visible to the dogs, 149 cm away from them.

The dogs were held by their caregivers on the dog’s side of the room. A brown plastic bucket (20 cm high, 15 cm diameter) was placed on the floor in front of each tunnel, on the dog’s side of the room. The experimenter could only see the bucket at the other end of the tunnel containing the transparent screen, but the tunnel containing the opaque screen blocked her visual access to the second bucket on the dog’s side of the room. From their starting position, dogs could see both buckets and tunnels but, owing to the orientation of the tunnels, not the type of screen (transparent or opaque) contained in each tunnel (figure 1c,d). The distance between the closest corners of the tunnels was 113 cm, while the experimenter was 75 cm away from the middle of each tunnel (electronic supplementary material, figure S2).

At the beginning of each trial, the experimenter ensured to catch the dogs’ attention and to establish eye contact with them before calling the dogs’ names, making a surprised facial expression [48] and alternating gaze three times between the dog and one of the tunnels. At the end of the third gaze shift, the experimenter held her gaze for 10 s in the direction of the target object on the other side of the tunnel (figure 1c,d). After the 10 s had elapsed, the experimenter looked down and gave a verbal signal to the caregivers that they could release their dogs. The experimenter remained immobile for 30 s while the dog was free to move around the room.

Dogs were tested in four trials: two in which the experimenter looked through the opaque screen and two in which she looked through the transparent one. The screen (transparent or opaque) the experimenter looked through on the first trial was counterbalanced across subjects. The direction in which the experimenter looked (left or right) was pseudo-randomized across dogs by following ABBA, ABAB, BABA or BAAB patterns. Moreover, the colour of the tunnel (blue or yellow) containing the transparent screen and the position (left or right) of the transparent screen were counterbalanced across subjects.

After each test trial (except for the last one), a motivation trial was performed. In motivation trials, the experimenter took the buckets, left the room, placed pieces of food in both buckets outside of the dog’s view, entered the room and placed the buckets on the floor by the door. Dogs were allowed to eat the food. While the experimenter left the room with the buckets, the caregivers recalled their dog and assumed the initial position again, holding their dog in front of them. The experimenter re-entered the room and placed the (now) empty buckets in front of the tunnels before continuing with the next test trial.

#### 2.1.4 Statistical analyses

All statistical analyses were conducted in R [49], v. 4.2.2. Unless differently specified, the following analyses were pre-registered. A second coder scored the data of *ca* 30% of the subjects. Inter-observer reliability was excellent for all coded variables: first approached screen during manipulation check (Cohen’s *κ* = 1, *n* = 10, *p* = 0.002); first look (*κ* = 0.90, *n* = 40, *p* < 0.001); first choice (Cohen’s *κ* = 1, *n* = 43, *p* < 0.001); duration of looking in the direction of the transparent (Spearman correlation: *r*_S_ = 0.93, *n* = 44, *p* < 0.001) and opaque screen (Spearman correlation: *r*_S_ = 0.89, *n* = 44, *p* < 0.001); inspection behind tunnels (Cohen’s *κ* = 0.81, *n* = 33, *p* < 0.001).

##### 2.1.4.1 Manipulation check analysis

In an exploratory analysis, we counted the number of dogs that, upon being released, approached first the tunnel containing the transparent screen. We compared this number with the chance level using a binomial test.

##### 2.1.4.2 Test phase analysis

We calculated the proportion of time dogs spent looking at the gaze-congruent target (the side of the room looked at by the experimenter) over their total duration of looking time to both targets. Additionally, we scored whether the dogs’ first look and first choice in each trial were congruent with the experimenter’s gaze cue or not. Both of the latter variables were analysed as previous research on gaze following in dogs suggests that the subjects’ gaze and active choice need to be considered as separate variables [46]. We fitted a generalized linear mixed model (GLMM) [50] with beta error structure and logit link function [51,52] for the proportion response variable and GLMMs with binomial error structure and logit link function for the binary response variables [53] (for additional details, see electronic supplementary material).

In all models, we included condition (the experimenter looked through the opaque or transparent screen) as the only test predictor. Control predictors with fixed effect were the colour of the tunnel containing the transparent screen (blue or yellow), trial number (1–4), as well as the dogs’ age and sex. Finally, we included the random slopes of the condition and trial number within the subject (the correlations between random slopes and intercept were pruned in case of convergence issues).

The covariates trial number and age were *z*-transformed and, prior to entering the random slope part of the model, the condition was manually dummy coded and centred. To assess the significance of the individual fixed effects, we used the function *drop1* with argument ‘test’ set to ‘Chisq’, which runs likelihood ratio tests based on the comparison of the full model to reduced models, each lacking one fixed effect.

In an exploratory analysis, we compared the dogs’ probability to direct their first look to the gaze-congruent side of the room and their probability to choose the gaze-congruent bucket first to chance level (0.5), by conducting two *t*-tests on the data aggregated across trials. We adjusted the resulting *p*values for multiple comparisons using Bonferroni’s correction.

### 2.2 Results

#### 2.2.1 Manipulation check performance

Five dogs were excluded from the study because they did not pass the manipulation check phase (i.e. they failed to look through the transparent tunnel in both trials). Out of the remaining 32 (tested) dogs, 23 approached the transparent screen first on their first manipulation check trial (binomial test: *p* = 0.020, 95% confidence interval = 0.53−0.86). Even including the five dogs that did not pass the manipulation check phase, dogs as a group significantly preferred to approach first the tunnel containing the transparent screen within two manipulation check trials (*p* = 0.003, 95% confidence interval = 0.59−0.88).

#### 2.2.2 Proportion of looking time to the gaze-congruent target

The experimenter’s gaze cue (through the transparent or opaque screen) had no significant influence on the proportion of time dogs spent looking at the side of the room containing the cued bucket (electronic supplementary material, table S3). A significant main effect of sex showed that male dogs looked longer than females to the gaze-congruent object ($\chi_{1}^{2}=4.1$, *p* = 0.043), irrespective of whether the object was visible to the experimenter or not. None of the other control predictors explained the variance in the response significantly.

We confirmed the absence of a significant condition effect using a paired *t*-test based on mean values per subject and condition (*t* = −0.45, d.f. = 31, *p* = 0.659, Bayes factor: 0.21). An equivalence test (paired *t*-test assuming an equivalence bound of ±0.25) provided evidence that the dogs’ performance was the same across the two conditions (*t* = 2.05, d.f. = 31, *p* = 0.02).

#### 2.2.3 First look and first choice

On average, dogs looked first at the gaze-congruent side of the room on 60% (s.e.: ± 5%) of the trials, which was significantly above the chance level (*t* = 2.75, d.f. = 31, *p*_Bonferroni_ = 0.020). On average, dogs chose the gaze-congruent bucket on 56% of trials (s.e.: ± 3%), which was not significantly different from the chance level (*t* = 2.02, d.f. = 31, *p*_Bonferroni_ = 0.104).

**Figure 2:**
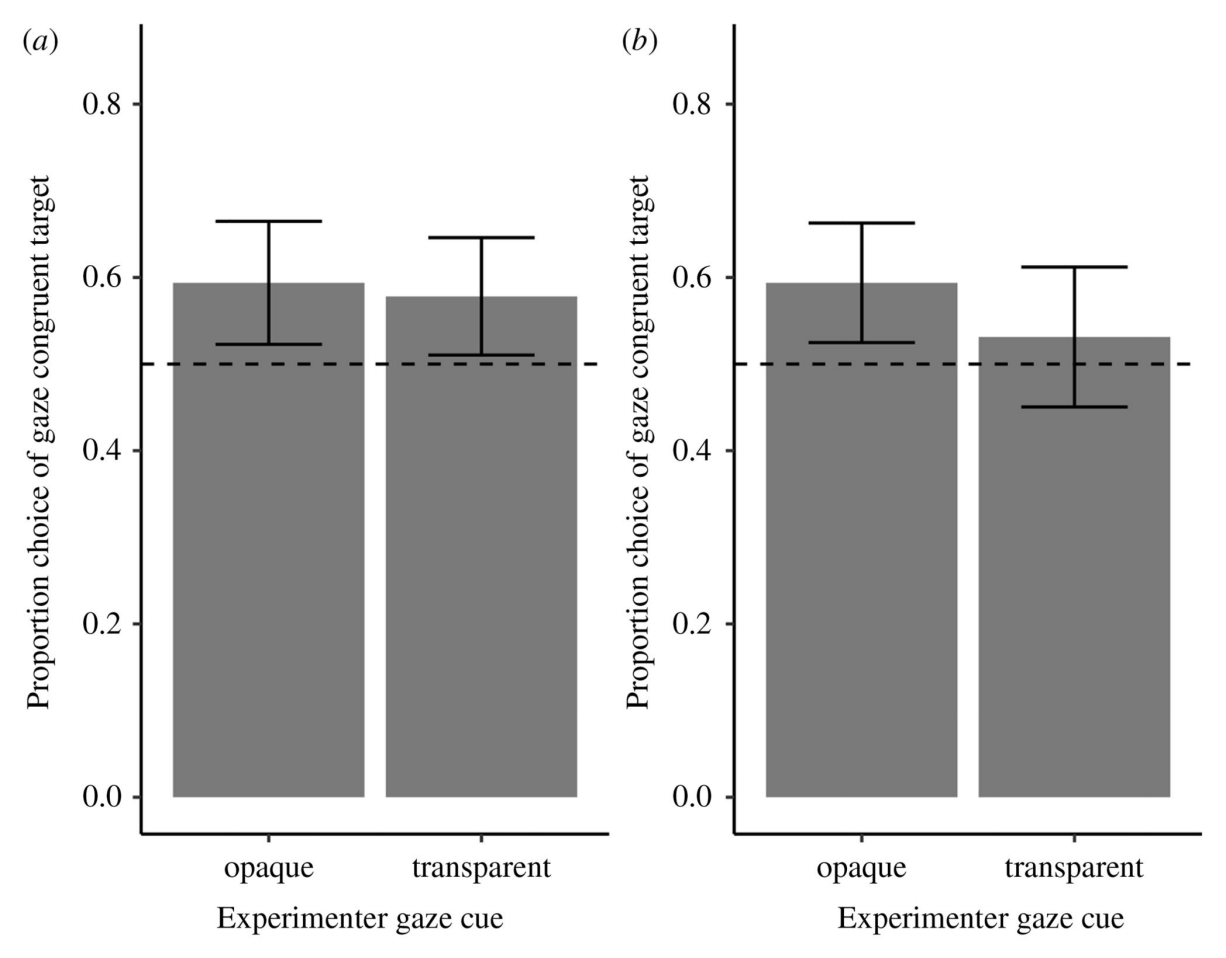
Experiment 1: the dogs’ first looks (*a*) and first choices (*b*). The bars represent mean values across dogs and trials in each condition and the error bars represent standard errors. The black dashed line represents the chance level (0.5).

Whether the experimenter looked through the transparent or opaque screen did not influence dogs’ first-look direction ($\chi_{1}^{2}=0.13$, *p* = 0.715; ΔAIC: 1.87, the reduced model without screen type had a lower Akaike information criterion (AIC) value than the full model; electronic supplementary material, table S4; figure 2a) or their first-approached container significantly ($\chi_{1}^{2}=3.21$, *p* = 0.073; electronic supplementary material, table S5; figure 2b) even though the inclusion of transparent/opaque screen did improve the model fit somewhat in the first choice analysis (ΔAIC: 1.21, the full model had a lower AIC value than the reduced model without screen type). Male dogs looked first to the gaze-congruent side of the room and approached first the gaze-congruent container more than females (first look: $\chi_{1}^{2}=4.1$, *p* = 0.045; first approach: $\chi_{1}^{2}=3.92$, *p* = 0.048). This suggests that they followed the experimenter’s gaze more than females, irrespective of whether for the experimenter the container was visible or not.

### 2.3 Discussion

The first experiment aimed to test whether dogs could infer whether humans have visual access to a target object, based on dogs’ own experience with novel visual barriers, in a gaze-following task. The results of the manipulation check indicate that the experience phase was effective in teaching the dogs the location or the colour of the tunnel containing the transparent screen. Indeed, the majority of dogs approached first the transparent screen when they themselves wanted to watch the experimenter on the other side of the room.

Nevertheless, whether the experimenter’s gaze was blocked by an opaque barrier or not did not influence the dogs’ gaze following during the test. Therefore, it seems that dogs recalled from which tunnel they themselves could gain visual access to the opposite side of the room during the experience phase. However, during test trials, either they did not attribute visual access to the experimenter or their gaze-following behaviour was anyhow unaffected by whether the experimenter could actually see or not the bucket she turned her head towards. The gaze-following results did not change when analysing only the data of the 23 dogs that passed the manipulation check on their first trial (see electronic supplementary material, tables S14 and S15). The lack of differential behaviour between test conditions is consistent with the behaviour of chimpanzees in a comparable task [16] but inconsistent with that of human 18 month olds [15].

Overall, the results of our first experiment are consistent with those of MacLean *et al*. [54], who found that, in a pointing task, dogs are sensitive to a human experimenter’s body orientation but they do not consider whether the experimenter’s view of a target is obstructed or not by opaque or transparent barriers. Indeed, in our experiment, dogs took into account the experimenter’s head direction, as evidenced by their first looks following the experimenter’s head direction above chance level. However, overall, their gaze-following behaviour was not modulated by whether the experimenter could see the target or not. Dogs’ absolute looking time to the transparent screen was longer but their looking time to the opaque screen only tended to be shorter, when the experimenter looked through the transparent rather than through the opaque screen.

Hence, similarly to MacLean *et al*. [54], we conclude that while it seems plausible that dogs are able to take a human’s perspective [28,31,35,38,39] and are able to follow a human’s gaze [46–48], they might have difficulties integrating the two skills.

One mentalistic possibility to explain how dogs might attribute visual access to others but still show no differential behaviour between the test conditions of this task is that dogs might attribute knowledge (derived from the experimenter having seen the buckets in the past) to the experimenter [28,31]. Indeed, the experimenter was the one placing the buckets in their position prior to each trial and therefore, while not able to see the bucket behind the opaque barrier at the moment of cueing, she was aware of its presence and might have been referring to it with her gaze cues. The fact that, in the foraging context of the present experiment, dogs followed the experimenter’s gaze even behind the opaque barrier is consistent with the literature [47].

## 3 Experiment 2

While the cooperative or competitive nature of tasks might influence apes’ performance (e.g. [22,55,56]), evidence for dogs’ perspective-taking capability comes from both cooperative (e.g. [28]) and ‘agonistic’ [26,32,37,39] situations. In experiment 2, we asked whether dogs would be more motivated to steal forbidden food by taking into account the experimenter’s visual access in a competitive scenario (similarly to the second experiment by [16]). It is possible that the dogs did not consider the experimenter’s visual access in the first experiment as relevant because in both cases they could explore both containers without the experimenter intervening. In experiment 2, the experimenter’s visual access should be more relevant given that pre-tests established that the experimenter would prevent the dogs from obtaining the food if she saw them approach it.

Subjects in experiment 2 belonged to breeds classified by Heberlein *et al*. [37] as having an ‘independent’ or ‘family’ working style, or to breeds within the same Fédération Cynologique Internationale (FCI) subgroups. We chose to only test these as independent and family breed groups were found to be more likely to take their owner’s perspective in a stealing task [37]. We hypothesized that dogs were able to project their own previous experience with the tunnels onto an experimenter in a competitive task. Hence, when forbidden from eating food, we predicted that they would try to steal it first from behind the tunnel containing the opaque rather than the transparent screen.

### 3.1 Methods

Experiment 2 was pre-registered: https://osf.io/nc9au/?view_only=c1464511e1774fa5b1373045d32e8179. The sample size was determined based on a power analysis (details in the pre-registration).

#### 3.1.1 Subjects

Subjects were 60 pure-bred pet dogs (33 females; electronic supplementary material, table S2, reports their demographic information). None of the dogs that participated in experiment 1 participated in experiment 2.

An additional 23 subjects were recruited but not included in the final sample. Specifically, the experiment was aborted owing to dogs showing emerging stress signals (*n* = 9) or failing to meet the inclusion criteria (*n* = 10). Additionally, four dogs were excluded owing to experimental error (*n* = 3) or the caregiver not following the instructions (*n* = 1).

#### 3.1.2 Set-up

Experiment 2 was conducted in the same room and with the same set up as experiment 1. However, the experimenter (V.S.) did not wear a mask covering her nose and mouth during tests and the buckets were replaced by white plastic plates. The distance between the closest corners of the tunnels was 50 cm; it was determined such that the experimenter could reach the plates in front of the tunnels simultaneously. Moreover, a curtain was mounted between the two tunnels during test trials to occlude the experimenter.

#### 3.1.3 Procedure and design

The whole procedure required a single session of approximately 60 min. The introduction phase was identical to the one described in experiment 1. Owing to the great inter-individual variability in dogs’ obedience in this task [57], similarly to Schwab & Huber [32], we excluded from our sample extremely obedient and extremely disobedient dogs by conducting a series of pre-tests (described in the electronic supplementary material).

##### 3.1.3.1 Experience phase

The tunnels were uncovered and the procedure used was similar to the one described for experiment 1, with the exception that now the owner showed food and a tennis ball above and in front of the screens. This change was made to avoid dogs receiving food from the experimenter.

##### 3.1.3.2 Test phase

In a within-subject design, dogs were tested in four trials: two in the test and two in the control conditions. At the beginning of each trial, dogs were held in the same starting position as during the pre-tests.

In the test condition, the experimenter gave the same verbal and gestural prohibition command as during pre-test trials, but then hid behind the curtain between the two tunnels and repeated the verbal command before the caregivers released their dogs. Hence, at the moment of choice, the dogs knew the experimenter was behind the curtain but could not see her themselves. If they remembered what they themselves were able to see during the experience phase, they should have realized the experimenter had potentially visual access to the plate behind the transparent screen but not to the plate behind the opaque one. Hence, if they understood they were not allowed to steal the food when observed by the experimenter, they should have preferentially approached the food hidden behind the opaque screen first.

In the control condition, the experimenter gave the same command as in the test condition but after walking behind the curtain, she left the room, visibly for the dogs. Hence, at the moment of choice, dogs should have not shown a preference for stealing one or the other piece of food, given that none of them was now visible to the (absent) experimenter.

Both types of trials lasted for a maximum of 2 min and could be repeated up to three times if a dog never even stole one piece of food. The conditions were presented in a blocked fashion and the condition presented first, the side and the colour of the tunnel containing the transparent screen were counterbalanced across subjects. After the first two trials (the first condition), the dog and the handler had a short break outside the testing room.

#### 3.1.4 Statistical analyses

As pre-registered, we excluded from the analyses trials in which dogs did not steal even one piece of food within the maximum trial duration of 2 min. This happened in three trials of the test condition of three different dogs. A second coder scored the data of 30% of the subjects. Inter-observer reliability was excellent both for the first choice (Cohen’s *κ* = 1, *n* = 71, *p* < 0.001) as well as for the latency to make a first choice (Spearman correlation: *r*_S_ = 0.99, *n* = 72, *p* < 0.001).

##### 3.1.4.1 First choice

To analyse the effect of condition (experimenter present or absent at the moment of choice) on dogs’ probability to steal the treat behind the opaque screen first, we fitted a GLMM with binomial error structure and logit link function. We included condition (experimental or control) as the only test predictor. Other control predictors with fixed effect were the order of presentation of conditions (factor with two levels: experimental or control first) and the trial number within the condition (1 or 2, *z*-transformed). Finally, we included the random intercept of subject ID and the random slope of condition (centred), to account for repeated observations of the same individuals and to allow for individual differences in the strength of the effect.

Second, we tested whether dogs’ probability to choose the treat behind the opaque screen first differed from chance level. To this end, we fitted two separate intercept-only models, one for the test and one for the control trials. We had pre-registered to analyse dogs’ probability to peek behind the opaque screen, in case they decided to do so. However, dogs never showed this behaviour.

##### 3.1.4.2 Latency

In an exploratory analysis, we assessed whether the dogs’ latency to steal the first treat was affected by our experimental manipulation, as done in previous studies using the stealing paradigm with dogs [26,32,39]. To this end, we fitted a GLMM with beta error structure and logit link function, using the function *glmmTMB* of the homonymous package. We divided the latencies (from the moment in which the dogs were released until the moment they made their first choice) by the maximum trial duration (2 min) to transform them into proportions.

We included as the only test predictor the interaction between the condition and the dogs’ first choice (steal from behind an opaque or transparent screen), as in the test condition, dogs might have been more hesitant to steal from behind the transparent than the opaque tunnel but this should have not been the case in the control condition. As control predictors, we included the trial number within the condition (1 or 2), *z*-transformed, and the order of presentation of conditions.

Finally, we included the random slope of the condition (manually dummy coded and centred) within the subject ID (random intercept). The significance of the fixed effect was evaluated with likelihood ratio tests (function drop1 with argument test set to ‘Chisq’). Because the interaction was not significant ($\chi_{1}^{2}=0.46$, *p* = 0.499), we dropped it from the model and investigated the significance of the main effects.

### 3.2 Results

#### 3.2.1 First choice

**Figure 3:**
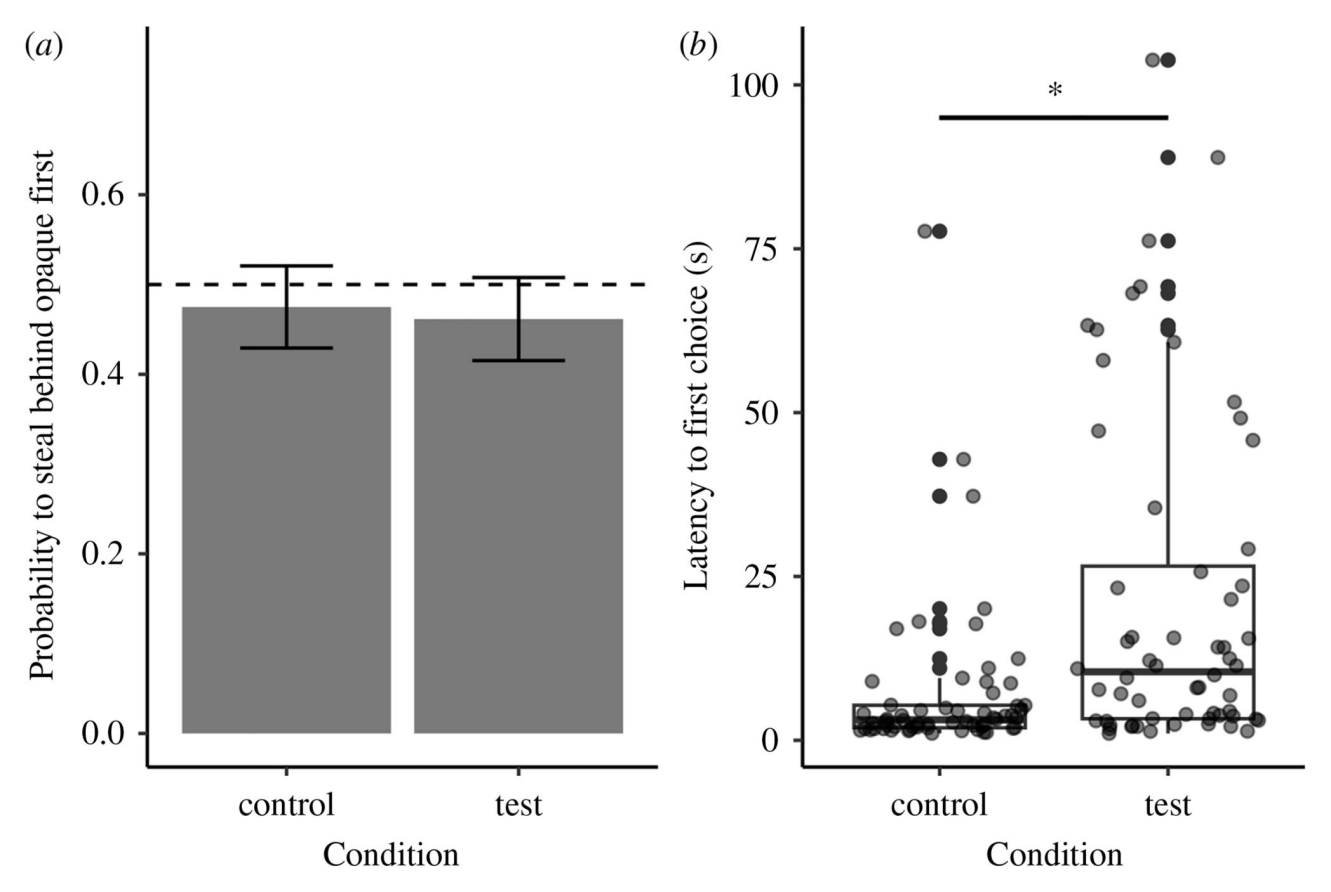
Experiment 2: (*a*) the dogs’ probability to steal the treat behind the opaque barrier first and (*b*) their latency (in seconds) to steal their first treat. (*a*) The bars represent mean values across dogs and trials in each condition and the error bars represent standard errors. The black dashed line represents the chance level (0.5). (*b*) The dots represent each subject’s mean latency to steal their first treat between the two trials in each condition. The significance asterisk represents the effect of condition (GLMM in electronic supplementary material, table S11; *p* < 0.001).

The dogs’ probability to steal the treat behind the opaque screen first did not differ between conditions ($\chi_{1}^{2}=0.08$, *p* = 0.773; ΔAIC: 1.92, the reduced model without condition had a lower AIC value than the full model; figure 3*a*); neither the order of presentation of the conditions nor the trial number influenced dogs’ choices (electronic supplementary material, table S9). Moreover, dogs’ probability to steal the treat behind the opaque screen first did not significantly deviate from chance in any of the conditions (intercept-only model test: *z* = −0.76, *p* = 0.45; intercept-only model control: *z* = −0.53, *p* = 0.599).

#### 3.2.2 Latency

Dogs were slower to steal the first treat when the experimenter was present in the room compared with when she was absent, irrespective of which treat they chose to steal first ($\chi_{1}^{2}=18.64$, *p* < 0.001; ΔAIC: 16.64, the full model had a lower AIC value than the reduced model without condition; figure 3b and electronic supplementary material, table S11).

### 3.3 Discussion

With experiment 2, we aimed to test whether dogs, in a competitive context involving forbidden food, would be more motivated to infer the experimenter’s visual access. The dogs did not prefer to steal a forbidden treat first from a location that afforded geometrical occlusion from the experimenter’s perspective but not from their own.

An exploratory analysis (see electronic supplementary material) revealed that terriers preferred stealing from behind the opaque barrier more than other breed groups, irrespective of the experimenter’s presence, a possible by-product of their artificial selection for underground hunting, which might lead them to prefer shielded or enclosed spaces. While their preferential first approach of the opaque screen indicates that terriers could remember the properties of the screens even when they were no longer visible to them, their preference is uninformative about their mind-reading ability, given that it was evident in both the experimental and control conditions.

## 4 General discussion

With this study, we adapted the methodology proposed by Heyes [14] to test whether dogs can infer what others can or cannot see, in a situation where behavioural and geometrical cues are not diagnostic and the only cue dogs can rely on to infer the mental state of ‘seeing’ is their own experience with novel visual barriers. Based on a previous study with 18-month-old humans [15], we had predicted that dogs would have followed more often a human experimenter’s gaze when her line of sight to a target was unobstructed compared with when it was obstructed. Additionally, based on previous studies with chimpanzees [16] and dogs [37], we had predicted that dogs would have been more likely to steal first a treat that was hidden from the experimenter’s line of sight, compared with a treat that was not. However, neither in the cooperative (gaze following) nor in the competitive (stealing) context did the dogs behave as if they were taking into account the human experimenter’s mental state of ‘seeing’.

A limitation in dogs’ perspective-taking abilities or secondary, task-related factors might account for this outcome. First, dogs might be able to reason about first-order (directly accessible) perceptual representations of objects, agents, barriers and the geometrical relations among them (e.g. [28,35,37]) but they might be unable to reason about others’ mental states (as suggested by [58], about ravens). Indeed, in our first experiment, dogs followed an experimenter’s gaze to a target above chance levels, irrespective of whether the experimenter could actually see the target or not. This result is consistent with the proposal that following others’ gaze might not require consideration of others’ mental states [59], even though there is evidence that dogs take into account contextual factors such as the location and size of occluding barriers [35] and the geometrical relationship between the agents and their visual target [28].

Also in our second experiment, the dogs did not take into account the experimenter’s visual access when choosing whether to steal first a treat that was visible to the experimenter or one that was not. Taken together, these results suggest that dogs do not possess or make use of the cognitive ability necessary to pass the goggles test in its current implementations, that is the ability to project their own past experiences onto others. In our versions of the goggles test, dogs’ performance might be better explained by the use of an egocentric strategy. In other words, the dogs in this study might have relied on the behavioural and contextual cues they could observe at that moment [38]. Such cues are the presence or absence of the experimenter in the room [26,32,44], the presence of light in the room [39], the visibility of the experimenters’ eyes [26,31], and the presence of an uninterrupted line of sight between the experimenter and the target [35,37,38].

Given dogs’ failure to take into account the experimenter’s visual access in the current tasks that, unlike previously used paradigms, require projecting their own previous experience onto others, one could assume that the perspective-taking abilities shown in those previous studies (reviewed in [20]) are not based on experience projection. This notion is consistent with some previous studies on dogs’ perspective-taking as well. For example, dogs did not consider an experimenter’s visual access when following pointing gestures [54] and they did not try to hide visual cues concerning their approach to forbidden food in a set up similar to that of our experiment 2. Namely, when the human experimenter who forbade them from eating the food was present but not visible at the moment of stealing [45].

Alternatively, dogs might be able to take into account others’ mental states but our experimental set ups were not suited for dogs to show this ability. To test whether our findings are owing to a lack of motivation, it would be interesting to replicate our experiments with the screens being directly visible to the dogs. Observing the expected pattern of results in such a setting could at least rule out that dogs are not motivated to take into account the experimenter’s perspective at all in these tasks.

It is important to note that, after stealing a piece of food, dogs could always steal the second one as well and, in the vast majority of cases, they did so. Moreover, we did not train the dogs to not steal. Rather, in short (20 s versus 1 min of [37]) pre-test trials, we selected dogs that would obey the experimenter and we did not intersperse ‘refreshment’ trials between test trials. These were instead used in previous studies using the stealing paradigm with dogs (e.g. [26]) to reinforce the idea that the experimenter was going to intervene when witnessing stealing attempts. Additionally, our test trials differed from our pre-test trials. Only during test trials did the experimenter hide behind a curtain. Dogs might have realized that the curtain now affected the experimenter’s ability to physically prevent them from stealing the treats, had she tried to do so. Hiding the experimenter from the dogs was, however, necessary to ensure that dogs based their decision on the experimenter’s inferred line of sight rather than on the observation of direct geometrical relations between the experimenter’s open eyes and an unobstructed line of sight to one of the treats [60]. Hence, it could be argued that our procedure might have decreased dogs’ motivation to carefully choose which treat to steal first, given that nothing happened during test trials if dogs chose the treat visible to the experimenter and dogs could eat both treats. However, in our pre-tests, we made sure to select food-motivated dogs with the appropriate obedience levels. Additionally, the dogs did react to our manipulations, i.e. the gaze cue (experiment 1) and, at least in their latency to steal, to the presence of the experimenter (experiment 2). Moreover, dogs’ behaviour was not affected by the trial number and an exploratory analysis (see electronic supplementary material) revealed that in the first trial, they did not distinguish between the experimental and control conditions either, making it unlikely that their overall performance was owing to the experimenter’s missing reinforcement in the test phase. Nevertheless, it is possible that dogs are sensitive to others’ visual access but the procedure used in this study did not motivate them enough to make use of such information.

The failure to show perspective-taking in these experiments might be owing to the novelty of the tunnels as visual barriers. A human selectively looking through a narrow tunnel might be an event dogs did not encounter previously in their lives. An effect of previous experience would reconcile well with the hypothesis that dogs’ perspective-taking abilities depend on their life history [33]. Despite the possibly artificial nature of our set up, it is important to note, however, that one of the requisites for the goggles test is that the animals have no previous experience with the visual barriers employed during the test, to exclude the possibility that they use previously learned behavioural rules to solve the task [12,14]. Moreover, the results of our manipulation check indicate that dogs, in a first-person context, could make use of the information they had acquired about the properties of the screens during the experience phase.

The fact that dogs hesitated more to steal a piece of food when the experimenter was present compared with when she left the room is consistent with previous literature showing that dogs are sensitive to the presence/absence of an experimenter in perspective-taking tasks (e.g. [26,32,44]). However, this result is uninformative about dogs’ mind-reading abilities as dogs’ latency was unaffected by which treat (the one behind the opaque or transparent screen) they chose to steal first. Moreover, dogs might have learned to rely on the presence/absence of the experimenter during the pre-test phase.

In conclusion, we have no evidence to suggest that dogs can reason about the mental state of ‘seeing’ when the only cue available to infer ‘seeing’ is their own previous visual experience with novel visual barriers. To explain dogs’ performance in this study a ‘Theory of Behaviour’, rather than a ‘ToM’, seems sufficient. However, it is too early to dismiss the possibility that task-related factors might explain the dogs’ failure to consider the experimenter’s visual perspective in the current gaze-following and stealing tasks.

## Supplementary Material

Supplementary Material

## Data Availability

All information for reproducing the analyses is publicly available here [61]. Supplementary material is available online [62].
