# Supplementary material for: Dogs do not use their own experience with novel barriers to infer others’ visual access
Source: Proc Biol Sci. Author manuscript; Available in PMC 2024 Jul 26. (PMC7616301; doi:10.1098/rspb.2023.2934)
Supplement: Supplementary file 1 [file EMS196925-supplement-Supplementary_Material.docx]

**Supplementary materials for: Dogs do not use their own experience with novel barriers to infer others’ visual access**

Lucrezia Lonardo, Martina Putnik, Veronika Szewczak, Ludwig Huber* and Christoph J. Völter*

*shared senior authorship

Proceedings of the Royal Society B

Article DOI: 10.1098/rspb

Supplementary materials

# Supplementary text

## Procedure

### Experiment 1

During the experience phase, dogs were given the possibility to gain experience with the appearance of the room from the side where the experimenter would have been during the later test. Dog and experimenter (M.P.) were on opposite sides of the room, separated by a set of fences and the two tunnels (Fig. 1a-b). The experimenter attracted the unleashed dogs’ attention to one of the tunnels by showing a piece of food in front of (Fig. 1a) and above (Fig 1b) the tunnel four times. Then, she dropped the piece of food on the dogs’ side of the room, in a position equidistant from both tunnels. The same procedure was repeated for the second tunnel with a new piece of food. We counterbalanced across dogs the tunnel (left or right) to which the dogs’ attention was brought first. Next, the experimenter bounced a tennis ball on the floor first behind one screen and then a second ball behind the second screen. As with the piece of food, dogs received a tennis ball, at a position equidistant from the two tunnels, after the presentations at each tunnel. Lastly the experimenter poured dry food in a bowl four times in front of each screen. The dogs saw (and heard) the food and toys when they were shown in front of the tunnel containing the transparent screen. They did not see them (although they could hear them) when they were shown in front of the tunnel with the opaque screen. Next, the dogs and experimenter switched sides in the room, for a manipulation check.

### Experiment 2

During the pre-tests, both tunnels were covered with blankets. Dogs started each trial by being held from their blindfolded caregiver 240 cm away from the tunnels.  

#### Pre-test I a)

With the first pre-test, we aimed at excluding dogs that could not refrain from eating prohibited food while being observed. The experimenter baited both plates at the same time and gave the dogs a verbal command to wait (e.g., “stay” or “no”) paired with a raised hand gesture.The verbal command was repeated a second time before the caregivers released their dogs. If dogs did not wait for 20 seconds (the duration of a trial) without approaching the plates, the experimenter immediately removed the plates (and the food on them), asked the caregivers to call their dogs back again and started a new trial. The pre-test continued for a maximum of 10 trials or until the dogs could wait for a whole trial without the experimenter having to intervene. If dogs did not wait for a whole trial within ten trials they were excluded from the study. 

#### Pre-test II

With the second pre-test, we aimed at excluding dogs that were too obedient and never tried to steal food even in the experimenter’s absence. The experimenter performed the same baiting procedure, verbal and gestural cue in front of the dogs but then left the room. Upon hearing the closing door, the caregivers released their dogs. Dogs had eight trials to start approaching the food within 20 seconds from being released in at least one trial. If dogs did not do so, they were excluded from the study.   

#### Pre-test I b)

Finally, we repeated pre-test I again in the same way as described above, to reiterate that dogs were not allowed to eat the food when the experimenter could see them. 

## Statistical Analyses

Initially all models included a parameter for the correlation between random intercepts and slopes, but these were removed when necessary to enable the models to converge.

### Experiment 1

#### Proportion of looking time to the gaze-congruent target and probability to look first at and choose first the gaze-congruent target

All models with beta error structure were fitted in R using the function *glmmTMB* of the homonymous package. We transformed the response so as to exclude the exact values of 0 and 1, as the beta distribution is not defined for these extremes (Smithson & Verkuilen, 2006). We checked that the beta model was not overdispersed (dispersion parameter: 0.71) and that all variance inflation factors (VIFs) were close to 1, using the function *vif* of the package *car* on a linear model. The same procedure was used to assess VIFs for the first choice and first look models. 

We additionally explored if the results would change if we used a stricter inclusion criterion. Hence, we repeated the analyses of the proportion of time dogs spent looking to the gaze-congruent target and of dogs’ first look, in the same ways as described in the main text, but this time only including the 23 dogs that passed the manipulation check on their first trial.

Binomial models (to analyse dogs’ first look and first choice) were fitted using the R function *lmer* of the package *lme4* (Bates et al., 2015). The models had the same structure as described in the main text and included correlation parameters between random intercept and slopes.

#### Absolute looking time in the direction of the tunnels

As pre-registered, we analysed the dogs’ looking time in the direction of the tunnel containing the transparent screen with a LMM (Baayen, 2008). As the original response was skewed, we used a square root transformation. The model had the same test and control predictors as the one used to analyse the proportion of looking time to the gaze-congruent target, described in the main text. 

Additionally, in an exploratory analysis, we assessed whether the dogs’ absolute looking time to the tunnel containing the opaque screen too was influenced by condition. Hence, we fitted a second LMM to this response variable (square-root transformed due to a skewed distribution), with the same structure as the model analysing the absolute looking times to the tunnel containing the transparent screen. In both cases, to draw inference about the single fixed effects, we re-fitted the models with restricted maximum likelihood using the function *lmer* of the package *lmerTest.* 

#### Probability to look in the direction of the transparent screen at least once during a trial

We analysed whether the dogs’ probability to look in the direction of the tunnel containing the transparent screen at least once during a trial depended on the experimenter’s looking direction. To this end, we fitted a binomial GLMM (Baayen, 2008) with logit link function (McCullagh & Nelder, 1989) and with the same structure as the models above. 

#### Probability of inspections behind the opaque tunnel

To analyse the effect of the type of screen the experimenter looked through (opaque or transparent) on dogs’ probability to walk around and inspect the area behind the opaque tunnel first (Fig. S1), we fitted a GLMM with binomial error structure and logit link function. The same predictors and random effect structure as described above were included in this model. Only trials in which the dog inspected the area behind at least one of the tunnels were included in this analysis.  

### Experiment 2

#### First choice – terriers

We explored the possibility that the dogs’ breed group might have affected their stealing behaviour. Indeed, previous evidence from experimental perspective-taking contexts (Heberlein et al., 2017; Lonardo et al., 2021) as well as from owner-reports (Morrill et al., 2022) suggests that these breeds might behave differently from others when following human cues. We specifically asked whether belonging to the terrier group affected the probability of stealing the treat behind the opaque barrier first, because terriers constituted the largest subsample within the breeds tested in our experiment. We fitted a binomial GLMM with logit link function to analyse the dogs’ first choice in the two conditions. We included as fixed effects: whether the dog was a terrier or not and its interaction with the experimental condition (control or test); the trial number within condition (z-transformed); and the order of presentation of conditions. The random slope of condition (centred) within subject was included as well. Because the interaction was not significant (likelihood ratio test: χ^2^_1_=1.99, *p*= 0.158), we dropped it from the model to be able to interpret the individual fixed effects. 

#### First choice – first trial only

To investigate whether our testing procedure might have made the dogs less likely to take into account the experimenter’s visual perspective, given that during test trials the experimenter never prevented dogs from eating any of the treats, we ran an exploratory analysis of the first trial only (between-subjects design with regards to the experimental condition). 

To the dogs’ first choice during the first trial, we fitted a binomial model similar to the one described in the main text, with the exception that we did not include trial number and the order of presentation of the conditions as predictors. Moreover, we did not include the random slope of condition nor the random intercept of subject.  The model was fitted using the function *glm*. 

## Results 

### Experiment 1

#### Proportion of looking time to the gaze-congruent target and probability to look first at the gaze-congruent target

The main results were not affected if only the 23 dogs that passed the manipulation check on their first trial were included in the analyses. Indeed, these dogs’ proportion of looking time to the gaze-congruent target and their probability to look first at the gaze-congruent target did not differ between conditions (Tables S14 and S15).

#### Absolute looking time in the direction of the tunnels

Dogs looked longer in the direction of the tunnel containing the transparent screen when the experimenter looked through the transparent compared to when she looked through the opaque screen (t _91.21_ = 2.48, *p* = .016; Table S6). Moreover, they tended to look for a shorter time in the direction of the tunnel containing the opaque screen when the experimenter looked through the transparent compared to through the opaque screen, but this difference did not reach statistical significance (t _91.19 _= -1.97, *p* = .055; Table S7). 

#### Probability to look in the direction of the transparent screen at least once during a trial

Dogs were more likely to look at least once during a trial in the direction of the transparent screen, when the experimenter had also looked through the transparent compared to the opaque screen (χ^2^_1_ = 4.94, *p* = .026; Table S8). The estimates of this model were quite unstable (i.e., varied considerably) depending on which subject was excluded from the analysis. 

#### Probability of inspections behind the opaque tunnel

The probability that dogs inspected the area behind the opaque tunnel was not influenced by the experimenter’s looking behaviour (χ^2^_1 _=1.88, *p* = .171; Table S9). Older dogs were less likely than younger ones and males tended to be less likely than females to inspect the area behind the opaque tunnel (age: χ^2^_1 _= 5.22, *p* = .022; sex: χ^2^_1 _= 3.77, p =.052), irrespective of condition. 

###  Experiment 2

#### First choice – terriers

The proportion of dogs stealing behind the opaque barrier, irrespective of condition, was significantly higher among terriers compared to all other breed groups (χ^2^_1_= 9.51, *p*= .002; Table S12). Hence, terriers showed a significant preference to steal the treat behind the opaque barrier first but their choice was not influenced by whether the experimenter was present or absent in the room. 

#### First choice – first trial only

Already from the first trials, dogs did not prefer to steal first the treat behind the opaque barrier (Wald test of the effect of condition: z= 0.26, *p*= .796; Table S13). 

References

Baayen, R. H. (2008). *Analyzing Linguistic Data: A Practical Introduction to Statistics Using R*. Cambridge University Press. https://doi.org/10.1017/CBO9780511801686

Heberlein, M. T. E., Turner, D. C., & Manser, M. B. (2017). Dogs’ (Canis familiaris) attention to human perception: Influence of breed groups and life experiences. *Journal of Comparative Psychology*, *131*(1), 19–29. https://doi.org/10.1037/com0000050

Lonardo, L., Völter, C. J., Lamm, C., & Huber, L. (2021). Dogs follow human misleading suggestions more often when the informant has a false belief. *Proceedings of the Royal Society B: Biological Sciences*, *288*(1955), 20210906. https://doi.org/10.1098/rspb.2021.0906

Morrill, K., Hekman, J., Li, X., McClure, J., Logan, B., Goodman, L., Gao, M., Dong, Y., Alonso, M., Carmichael, E., Snyder-Mackler, N., Alonso, J., Noh, H. J., Johnson, J., Koltookian, M., Lieu, C., Megquier, K., Swofford, R., Turner-Maier, J., … Karlsson, E. K. (2022). Ancestry-inclusive dog genomics challenges popular breed stereotypes. *Science*, *376*(6592), eabk0639. https://doi.org/10.1126/science.abk0639

Tables

Table S1

Demographic information of the dogs in Experiment 1

| Subject ID | Sex | Age (months) | Breed |
| --- | --- | --- | --- |
| 01 | F | 79 | Giant Schnauzer |
| 02 | F | 56 | Miniature Poodle |
| 03 | F | 43 | Australian Shepherd |
| 04 | F | 62 | Mix (Dachsund) |
| 05 | M | 28 | Mix |
| 06 | F | 94 | Whippet |
| 07 | F | 68 | Rough Collie |
| 08 | F | 103 | Labrador Retriever |
| 09 | F | 16 | Whippet |
| 10 | F | 90 | Yorkshire Terrier |
| 11 | F | 131 | Whippet |
| 12 | M | 84 | Smooth Collie |
| 13 | F | 89 | Mix (Austrian Pinscher) |
| 14 | F | 100 | Airedale Terrier |
| 15 | M | 14 | Labrador Retriever |
| 16 | F | 9 | Miniature Pinscher |
| 17 | M | 45 | Mix (Puli) |
| 18 | M | 140 | Labradoodle |
| 19 | F | 56 | Mix (Pumi) |
| 20 | F | 102 | Mix (Jack Russel, Malinois) |
| 21 | F | 121 | Beagle |
| 22 | M | 65 | Pyrenean Sheep Dog |
| 23 | M | 99 | English Setter |
| 24 | M | 46 | Australian Shepherd |
| 25 | F | 74 | Flat Coated Retriever |
| 26 | M | 162 | Pyrenean Sheep Dogs |
| 27 | F | 49 | Hungarian Vizsla |
| 28 | F | 168 | Mix (Pitbull) |
| 29 | F | 154 | Chihuaua |
| 30 | F | 30 | Airedale Terrier |
| 31 | F | 54 | Whippet |
| 32 | F | 32 | Australian Shepherd |

Table S2

Demographic information of the dogs in Experiment 2

| Subject ID | Sex | Age (months) | Breed |
| --- | --- | --- | --- |
| 01 | F | 119 | Irish Glenn of Imaal Terrier |
| 02 | M | 141 | Hovawart |
| 03 | M | 68 | French Bulldog |
| 04 | M | 93 | Irish Glenn of Imaal Terrier |
| 05 | M | 62 | Bernese Mountain Dog |
| 06 | M | 84 | Rottweiler |
| 07 | F | 107 | Whippet |
| 08 | M | 64 | Border Terrier |
| 09 | M | 89 | American Staffordishire Terrier |
| 10 | F | 22 | Wirehaired Dachsund |
| 11 | F | 109 | Irish Glenn of Imaal Terrier |
| 12 | F | 54 | Shih Tzu |
| 13 | F | 81 | Maltese |
| 14 | F | 49 | Irish Glenn of Imaal Terrier |
| 15 | M | 47 | Siberian Husky |
| 16 | M | 17 | Whippet |
| 17 | M | 50 | Greyhound |
| 18 | M | 78 | French Bulldog |
| 19 | F | 15 | Siberian Husky |
| 20 | F | 16 | Border Terrier |
| 21 | F | 90 | Samoyed |
| 22 | F | 73 | Whippet |
| 23 | F | 17 | Miniature Poodle |
| 24 | M | 89 | Greater Swiss Mountain Dog |
| 25 | F | 56 | Parson Russel Terrier |
| 26 | F | 90 | Chihuahua |
| 27 | F | 16 | Miniature Poodle |
| 28 | M | 52 | American Staffordshire Terrier |
| 29 | F | 85 | Border Terrier |
| 30 | F | 47 | West Highland White Terrier |
| 31 | F | 69 | Whippet |
| 32 | F | 29 | Hovawart |
| 33 | F | 55 | Parson Russel Terrier |
| 34 | M | 27 | Staffordshire Bull Terrier |
| 35 | F | 74 | Havanese |
| 36 | F | 108 | Border Terrier |
| 37 | F | 18 | Little Lion Dog |
| 38 | M | 14 | Whippet |
| 39 | M | 24 | Havanese |
| 40 | M | 54 | Miniature Bull Terrier |
| 41 | M | 123 | Whippet |
| 42 | F | 105 | Bernese Mountain Dog |
| 43 | F | 58 | Parson Russel Terrier |
| 44 | F | 17 | Miniature Dachshund |
| 45 | F | 20 | Whippet |
| 46 | M | 75 | Hovawart |
| 47 | M | 60 | Bedlington Terrier |
| 48 | M | 20 | Fox Terrier |
| 49 | M | 21 | Parson Russel Terrier |
| 50 | M | 67 | Parson Russel Terrier |
| 51 | M | 77 | Petit Brabancon |
| 52 | M | 18 | Shih Tzu |
| 53 | F | 128 | West Highland Terrier |
| 54 | F | 25 | Maltese |
| 55 | M | 108 | Whippet |
| 56 | F | 42 | Bernese Mountain Dog |
| 57 | M | 14 | Cocker Spaniel |
| 58 | F | 60 | Siberian Husky |
| 59 | F | 11 | Havanese |
| 60 | F | 26 | Boston Terrier |

Table S3

Experiment 1 – results of the beta model investigating the effect of condition (whether the experimenter gazed through the transparent or opaque screen) on dogs’ proportion of looking time to the gaze-congruent target

|  | Estimate | Std. Error | χ^2^ | Df | *p* | CI min | CI max | min | max | delta AIC |
| --- | --- | --- | --- | --- | --- | --- | --- | --- | --- | --- |
| Intercept | 0.22 | 0.26 | ^(1)^ | ^(1)^ | ^(1)^ | -0.33 | 0.72 | 0.13 | 0.3 | ^(1)^ |
| Condition^(2)^ | -0.13 | 0.38 | 0.12 | 1 | .724 | -0.83 | 0.6 | -0.28 | 0 | 1.88 |
| Colour of tunnel^(3)^ | 0.09 | 0.21 | 0.17 | 1 | .682 | -0.34 | 0.54 | 0.05 | 0.17 | 1.83 |
| Trial nr.^(4)^ | 0.13 | 0.11 | 1.24 | 1 | .265 | -0.1 | 0.35 | 0.08 | 0.18 | 0.76 |
| Age^(4)^ | 0.13 | 0.11 | 1.32 | 1 | .251 | -0.08 | 0.34 | 0.09 | 0.16 | 0.68 |
| Sex^(5)^ | 0.49 | 0.24 | 4.1 | 1 | .043 | 0 | 0.97 | 0.41 | 0.56 | -2.1 |

Note:  (1) Not reported due to its very limited interpretability

(2) Reference category: opaque

(3) Colour of the tunnel containing the transparent screen. Reference category: blue

(4) Z-transformed to a mean of 0 and standard deviation of 1

(5) Reference category: female

Table S4

|  | Estimate | Std. Error | χ^2^ | Df | *p* | CI min | CI max | min | max | delta AIC |  |
| --- | --- | --- | --- | --- | --- | --- | --- | --- | --- | --- | --- |
| Intercept | 0.19 | 0.54 | ^(1)^ | ^(1)^ | ^(1)^ | -1.39 | 4.46 | -0.04 | 0.44 | ^(1)^ |  |
| Condition^(2)^ | -0.28 | 0.78 | 0.13 | 1 | .715 | -4.5 | 3.35 | -0.55 | -0.07 | 1.87 |  |
| Colour of tunnel^(3)^ | 0.56 | 0.49 | 1.36 | 1 | .244 | -0.51 | 4.13 | 0.43 | 0.74 | 0.64 |  |
| Trial nr.^(4)^ | -0.3 | 0.26 | 1.35 | 1 | .246 | -3.45 | 0.28 | -0.48 | -0.22 | 0.65 |  |
| Age^(4)^ | 0.21 | 0.23 | 0.27 | 1 | .6 | -0.45 | 1.53 | 0.11 | 0.3 | 1.73 |  |
| Sex^(5)^ | 1.07 | 0.6 | 3.77 | 1 | .052 | -0.08 | 6.26 | 0.73 | 1.48 | -1.77 |  |

Experiment 1 – results of the binomial model investigating the effect of condition (whether the experimenter gazed through the transparent or opaque screen) on dogs’ first look

Note:  (1) Not reported due to its very limited interpretability

(2) Reference category: opaque

(3) Colour of the tunnel containing the transparent screen. Reference category: blue

(4) Z-transformed to a mean of 0 and standard deviation of 1

(5) Reference category: female

Table S5

Experiment 1 – results of the binomial model investigating the effect of condition (whether the experimenter gazed through the transparent or opaque screen) on dogs’ first choice

|  | Estimate | Std. Error | χ^2^ | Df | *p* | CI min | CI max | min | max | delta AIC |
| --- | --- | --- | --- | --- | --- | --- | --- | --- | --- | --- |
| Intercept | 0.43 | 0.59 | ^(1)^ | ^(1)^ | ^(1)^ | -1.25 | 10.93 | 0.24 | 0.71 | ^(1)^ |
| Condition^(2)^ | -0.31 | 1.5 | 3.21 | 1 | .073 | -29.32 | 12.56 | -0.74 | 0.08 | -1.21 |
| Colour of tunnel^(3)^ | 0.01 | 0.54 | 0 | 1 | 1 | -2.18 | 2.37 | -0.24 | 0.22 | 2 |
| Trial nr.^(4)^ | 0.31 | 0.29 | 1.13 | 1 | .289 | -0.77 | 5.16 | 0.16 | 0.41 | 0.87 |
| Age^(4)^ | 0.02 | 0.26 | 0.01 | 1 | .939 | -0.94 | 1.17 | -0.05 | 0.13 | 1.99 |
| Sex^(5)^ | 0.46 | 0.62 | 3.92 | 1 | .048 | -1.39 | 4.92 | 0.32 | 0.75 | -1.92 |

Note:  (1) Not reported due to its very limited interpretability

(2) Reference category: opaque

(3) Colour of the tunnel containing the transparent screen. Reference category: blue

(4) Z-transformed to a mean of 0 and standard deviation of 1

(5) Reference category: female

Table S6

Experiment 1 – results of the LMM investigating the effect of condition on the dogs’ absolute looking time to the tunnel containing the transparent screen.

|  | Estimate | Std. Error | t | Df | *p* | CI min | CI max | min^(6)^ | max^(6)^ |
| --- | --- | --- | --- | --- | --- | --- | --- | --- | --- |
| Intercept | 1.12 | 0.18 | ^(1)^ | ^(1)^ | ^(1)^ | 0.75 | 1.49 | 1.03 | 1.23 |
| Condition^(2)^ | 0.28 | 0.11 | 2.48 | 91.21 | .016 | 0.06 | 0.5 | 0.24 | 0.3 |
| Colour of tunnel^(3)^ | -0.29 | 0.22 | -1.31 | 28.34 | .225 | -0.74 | 0.14 | -0.4 | -0.21 |
| Trial nr.^(4)^ | -0.12 | 0.06 | -1.99 | 66.3 | .053 | -0.24 | -0.01 | -0.15 | -0.1 |
| Age^(4)^ | -0.03 | 0.11 | -0.26 | 28.4 | .807 | -0.26 | 0.21 | -0.09 | 0.03 |
| Sex^(5)^ | 0.06 | 0.25 | 0.26 | 28.32 | .823 | -0.41 | 0.55 | -0.06 | 0.14 |

Note:  (1) Not reported due to its very limited interpretability

(2) Reference category: opaque

(3) Colour of the tunnel containing the transparent screen. Reference category: blue

(4) Z-transformed to a mean of 0 and standard deviation of 1

(5) Reference category: female

(6) Min and Max report the range of estimates obtained when calculating model stability, i.e., when excluding each level of the random intercept at a time and fitting the model again. 

Table S7

Experiment 1 – results of the LMM investigating the effect of condition on the dogs’ absolute looking time to the tunnel containing the opaque screen.

|  | Estimate | Std. Error | t | Df | *p* | CI min | CI max | min^(6)^ | max^(6)^ |
| --- | --- | --- | --- | --- | --- | --- | --- | --- | --- |
| Intercept | 1.38 | 0.18 | ^(1)^ | ^(1)^ | ^(1)^ | 1.03 | 1.77 | 1.32 | 1.49 |
| Condition^(2)^ | -0.23 | 0.12 | -1.97 | 91.19 | .055 | -0.46 | -0.01 | -0.28 | -0.19 |
| Colour of tunnel^(3)^ | -0.2 | 0.22 | -0.92 | 28.04 | .395 | -0.63 | 0.27 | -0.32 | -0.11 |
| Trial nr.^(4)^ | -0.01 | 0.06 | -0.13 | 92.93 | .895 | -0.12 | 0.11 | -0.04 | 0.02 |
| Age^(4)^ | -0.03 | 0.11 | -0.32 | 28.01 | .763 | -0.26 | 0.19 | -0.1 | 0.06 |
| Sex^(5)^ | 0.14 | 0.24 | 0.6 | 28.04 | .581 | -0.34 | 0.61 | -0.06 | 0.28 |

Note:  (1) Not reported due to its very limited interpretability

(2) Reference category: opaque

(3) Colour of the tunnel containing the transparent screen. Reference category: blue

(4) Z-transformed to a mean of 0 and standard deviation of 1

(5) Reference category: female

(6) Min and Max report the range of estimates obtained when calculating model stability, i.e., when excluding each level of the random intercept at a time and fitting the model again. 

Table S8

Experiment 1 – results of the binomial GLMM investigating the effect of condition on the dogs’ probability to look to the tunnel containing the transparent screen at least once during a trial.

|  | Estimate | Std. Error | χ^2^ | Df | *p* | CI min | CI max | min^(6)^ | max^(6)^ |
| --- | --- | --- | --- | --- | --- | --- | --- | --- | --- |
| Intercept | 1.74 | 1.43 | ^(1)^ | ^(1)^ | ^(1)^ | -0.04 | 35.18 | 1.29 | 6.22 |
| Condition^(2)^ | 1.46 | 0.76 | 4.94 | 1 | .026 | -0.08 | 28.02 | 1.31 | 3.75 |
| Colour of tunnel^(3)^ | 0.25 | 1.3 | 0.04 | 1 | .848 | -11.26 | 13.92 | -0.33 | 0.86 |
| Trial nr.^(4)^ | -0.37 | 0.44 | 0.89 | 1 | .346 | -13.65 | 3.43 | -1.1 | -0.23 |
| Age^(4)^ | -0.82 | 0.78 | 1.49 | 1 | .222 | -22 | 1.16 | -1.06 | -0.59 |
| Sex^(5)^ | 1.17 | 1.44 | 0.63 | 1 | .428 | -8.35 | 31.45 | 0.72 | 1.6 |

Note:  (1) Not reported due to its very limited interpretability

(2) Reference category: opaque

(3) Colour of the tunnel containing the transparent screen. Reference category: blue

(4) Z-transformed to a mean of 0 and standard deviation of 1

(5) Reference category: female

(6) Min and Max report the range of estimates obtained when calculating model stability, i.e., when excluding each level of the random intercept at a time and fitting the model again. 

Table S9

Experiment 1 – results of the binomial GLMM investigating the effect of condition on the dogs’ probability to inspect behind the tunnel containing the opaque screen during a trial.

|  | Estimate | Std. Error | χ^2^ | Df | *p* | CI min | CI max | min^(6)^ | max^(6)^ |
| --- | --- | --- | --- | --- | --- | --- | --- | --- | --- |
| Intercept | 0.89 | 0.58 | ^(1)^ | ^(1)^ | ^(1)^ | -0.08 | 16.77 | 0.68 | 1.45 |
| Condition^(2)^ | -0.74 | 0.56 | 1.88 | 1 | .171 | -19 | 0.66 | -1.5 | -0.6 |
| Colour of tunnel^(3)^ | -0.58 | 0.55 | 1.18 | 1 | .278 | -12.59 | 1.26 | -1.01 | -0.39 |
| Trial nr.^(4)^ | -0.41 | 0.47 | 0.86 | 1 | .353 | -14.4 | 1.18 | -0.6 | -0.25 |
| Age^(4)^ | -0.71 | 0.37 | 5.22 | 1 | .022 | -10.22 | -0.15 | -1.01 | -0.47 |
| Sex^(5)^ | -1.76 | 1.19 | 3.77 | 1 | .052 | -27.59 | -0.71 | -2.38 | -0.81 |

Note:  (1) Not reported due to its very limited interpretability

(2) Reference category: opaque

(3) Colour of the tunnel containing the transparent screen. Reference category: blue

(4) Z-transformed to a mean of 0 and standard deviation of 1

(5) Reference category: female

(6) Min and Max report the range of estimates obtained when calculating model stability, i.e., when excluding each level of the random intercept at a time and fitting the model again. 

Table S10

Experiment 2 – results of the binomial GLMM investigating the effect of condition on the dogs’ probability to steal first the treat behind the opaque screen.

|  | Estimate | Std. Error | χ^2^ | Df | *p* | CI min | CI max | min^(5)^ | max^(5)^ | delta AIC |
| --- | --- | --- | --- | --- | --- | --- | --- | --- | --- | --- |
| Intercept | -0.25 | 0.34 | ^(1)^ | ^(1)^ | ^(1)^ | -0.98 | 0.42 | -0.35 | -0.16 | ^(1)^ |
| Condition^(2)^ | -0.09 | 0.3 | 0.08 | 1 | .773 | -0.75 | 0.59 | -0.19 | 0 | 1.92 |
| Trial nr. ^(3)^ | -0.11 | 0.15 | 0.56 | 1 | .456 | -0.42 | 0.18 | -0.16 | -0.07 | 1.44 |
| Order of conditions^ (4)^ | 0.24 | 0.44 | 0.28 | 1 | .594 | -0.65 | 1.2 | 0.14 | 0.34 | 1.72 |

Note:  (1) Not reported due to its very limited interpretability

(2) Reference category: control

(3) Z-transformed to a mean of 0 and standard deviation of 1

(4) Reference category: control first

(5) Min and Max report the range of estimates obtained when calculating model stability, i.e., when excluding each level of the random intercept at a time and fitting the model again. 

Table S11

Experiment 2 – results of the beta GLMM investigating the effect of condition on the dogs’ latency to steal the first treat.

|  | Estimate | Std. Error | χ^2^ | Df | *p* | CI min | CI max | min^(6)^ | max^(6)^ | delta AIC |
| --- | --- | --- | --- | --- | --- | --- | --- | --- | --- | --- |
| Intercept | -2.33 | 0.18 | ^(1)^ | ^(1)^ | ^(1)^ | -2.69 | -2.09 | -2.41 | -2.3 | ^(1)^ |
| Condition^(2)^ | 0.6 | 0.13 | 18.64 | 1 | < .001 | 0.34 | 0.87 | 0.57 | 0.63 | 1.44 |
| Choice ^(3)^ | -0.11 | 0.14 | 0.56 | 1 | .455 | -0.39 | 0.16 | -0.16 | -0.08 | -16.64 |
| Trial nr. ^(4)^ | -0.01 | 0.06 | 0.04 | 1 | .845 | -0.14 | 0.1 | -0.03 | 0 | 1.96 |
| Order of conditions^ (5)^ | 0.1 | 0.18 | 0.33 | 1 | .566 | -0.23 | 0.47 | 0.01 | 0.18 | 1.67 |

Note:  (1) Not reported due to its very limited interpretability

(2) Reference category: control

(3) Reference category: treat behind opaque screen 

(4) Z-transformed to a mean of 0 and standard deviation of 1

(5) Reference category: control first

(6) Min and Max report the range of estimates obtained when calculating model stability, i.e., when excluding each level of the random intercept at a time and fitting the model again. 

Table S12

Experiment 2 – results of the binomial GLMM investigating the effect of being a terrier on the dogs’ probability to steal the treat behind the opaque barrier first.

|  | Estimate | Std. Error | χ^2^ | Df | *p* | CI min | CI max | min^(6)^ | max^(6)^ |
| --- | --- | --- | --- | --- | --- | --- | --- | --- | --- |
| Intercept | -0.78 | 0.38 | ^(1)^ | ^(1)^ | ^(1)^ | -1.65 | -0.07 | -0.91 | -0.68 |
| Terrier^(2)^ | 1.4 | 0.45 | 9.51 | 1 | .002 | 0.62 | 2.47 | 1.27 | 1.56 |
| Condition ^(3)^ | -0.06 | 0.3 | 0.04 | 1 | .837 | -0.69 | 0.54 | -0.16 | 0.03 |
| Trial nr. ^(4)^ | -0.12 | 0.15 | 0.59 | 1 | .443 | -0.43 | 0.2 | -0.17 | -0.07 |
| Order of conditions^ (5)^ | 0.33 | 0.41 | 0.63 | 1 | .427 | -0.54 | 1.18 | 0.21 | 0.46 |

Note:  (1) Not reported due to its very limited interpretability

(2) Whether the dog was a terrier or not. Reference category: no 

(3) Reference category: control 

(4) Z-transformed to a mean of 0 and standard deviation of 1

(5) Reference category: control first

(6) Min and Max report the range of estimates obtained when calculating model stability, i.e., when excluding each level of the random intercept at a time and fitting the model again. 

Table S13

Experiment 2 – results of the binomial GLMM investigating the effect of being a terrier on the dogs’ probability to steal the treat behind the opaque barrier first.

|  | Estimate | Std. Error | z | *p* | CI min | CI max | min^(3)^ | max^(3)^ |
| --- | --- | --- | --- | --- | --- | --- | --- | --- |
| Intercept | 0 | 0.37 | ^(1)^ | ^(1)^ | -0.72 | 0.72 | -0.08 | 0.08 |
| Condition ^(2)^ | 0.13 | 0.52 | 0.26 | .796 | -0.88 | 1.16 | 0.05 | 0.21 |

Note:  (1) Not reported due to its very limited interpretability

(2) Reference category: control 

(3) Min and Max report the range of estimates obtained when calculating model stability, i.e., when excluding each level of the random intercept at a time and fitting the model again.

Table S14

Experiment 1 – results of the beta model investigating the effect of condition (whether the experimenter gazed through the transparent or opaque screen) on dogs’ proportion of looking time to the gaze-congruent target, including only the 23 dogs that passed the manipulation check on their first trial.

|  | Estimate | Std. Error | χ^2^ | Df | *p* | CI min | CI max | min | max |
| --- | --- | --- | --- | --- | --- | --- | --- | --- | --- |
| Intercept | 0.36 | 0.3 | ^(1)^ | ^(1)^ | ^(1)^ | -0.22 | 0.93 | 0.26 | 0.46 |
| Condition^(2)^ | -0.26 | 0.46 | 0.33 | 1 | .568 | -1.24 | 0.64 | -0.46 | -0.07 |
| Colour of tunnel^(3)^ | 0.06 | 0.26 | 0.06 | 1 | .81 | -0.49 | 0.54 | -0.03 | 0.11 |
| Trial nr.^(4)^ | 0.12 | 0.13 | 0.84 | 1 | .359 | -0.15 | 0.39 | 0.05 | 0.16 |
| Age^(4)^ | 0.14 | 0.14 | 0.94 | 1 | .333 | -0.13 | 0.41 | 0.09 | 0.21 |
| Sex^(5)^ | 0.29 | 0.33 | 0.78 | 1 | .377 | -0.37 | 0.92 | 0.21 | 0.37 |

Note:  (1) Not reported due to its very limited interpretability

(2) Reference category: opaque

(3) Colour of the tunnel containing the transparent screen. Reference category: blue

(4) Z-transformed to a mean of 0 and standard deviation of 1

(5) Reference category: female

Table S15

Experiment 1 – results of the binomial model investigating the effect of condition (whether the experimenter gazed through the transparent or opaque screen) on dogs’ first look, including only the 23 dogs that passed the manipulation check on their first trial.

|  | Estimate | Std. Error | χ^2^ | Df | *p* | CI min | CI max | min | max |
| --- | --- | --- | --- | --- | --- | --- | --- | --- | --- |
| Intercept | 0.32 | 0.59 | ^(1)^ | ^(1)^ | ^(1)^ | -1.85 | 7.39 | -0.01 | 0.66 |
| Condition^(2)^ | -0.53 | 0.86 | 0.23 | 1 | .628 | -11.36 | 4.4 | -0.82 | -0.24 |
| Colour of tunnel^(3)^ | 0.64 | 0.6 | 1.22 | 1 | .269 | -0.84 | 8.85 | 0.44 | 0.96 |
| Trial nr.^(4)^ | -0.53 | 0.31 | 3.31 | 1 | .069 | -8.76 | 0.13 | -0.62 | -0.42 |
| Age^(4)^ | 0.29 | 0.29 | 1 | 1 | .317 | -0.53 | 4.07 | 0.15 | 0.44 |
| Sex^(5)^ | 0.36 | 0.75 | 0.23 | 1 | .629 | -2.09 | 7.79 | -0.04 | 0.87 |

Note:  (1) Not reported due to its very limited interpretability

(2) Reference category: opaque

(3) Colour of the tunnel containing the transparent screen. Reference category: blue

(4) Z-transformed to a mean of 0 and standard deviation of 1

(5) Reference category: female

Figures

 Figure S1. A dog inspecting the area behind the tunnel containing the opaque screen.

Figure S2. A diagram showing an aerial view of the set up for Experiment 1. The blue and yellow rectangles indicate the tunnels and the brown circles indicate the buckets (only one was visible from the experimenter’s point of view, both were visible to the dog. Dogs were held by their caregiver, who was seated on a chair. Opposite to the dog, the experimenter knelt on the floor. The grey horizontal lines at the side of the tunnels indicate the barriers dividing the room in two sides (dog’s side and experimenter side). During the experience and manipulation check phases, an additional barrier was placed between the tunnels. The thick black line at the bottom of the figure indicates the position of the door to the room.
